# Identification and Expression Analysis of the *MLO* Gene Family Under Salt Stress in Cotton (*Gossypium hirsutum* L.)

**DOI:** 10.3390/life16030476

**Published:** 2026-03-16

**Authors:** Cong-Hua Feng, Junbo Zhen, Linlin Liu, Mengzhe Li, Mengmeng Jiang, Di Liu, Jina Chi

**Affiliations:** 1 Institute of Cotton, Hebei Academy of Agricultural and Forestry Sciences, Key Laboratory of Biology and Genetic Improvement of Cotton in Huanghuaihai Semiarid Area, Ministry of Agriculture and Rural Affairs, Hebei Key Laboratory of Cotton Bio-Breeding and Cultivation Physiology, Shijiazhuang 050051, China; fch19942024@163.com (C.-H.F.); zjb210@126.com (J.Z.); spp2016@126.com (L.L.); limengzhe0131@126.com (M.L.); jmm18270464849@163.com (M.J.); 2 Genetics Laboratory, College of Life Science, Hebei University, Baoding 071002, China

**Keywords:** *Gossypium hirsutum*, *GhMLO* gene family, transcriptome analysis, abiotic stress, gene expression

## Abstract

MLO (Mildew Resistance Locus O) genes encode seven-transmembrane proteins that function as critical regulators of powdery mildew resistance and abiotic stress responses. Despite their established importance, the *MLO* gene family in *Gossypium hirsutum* L. has not been systematically investigated under salt stress conditions. Here, we performed genome-wide identification of 46 *GhMLO* members using Hidden Markov Model and BLAST searches based on the latest cotton genome assembly. Phylogenetic analysis classified these genes into four distinct subfamilies. Transmembrane topology and conserved domain analyses revealed that all GhMLO proteins contain typical MLO domains and transmembrane structures, maintaining high structural similarity with dicotyledonous model plants. Synteny analysis demonstrated that the expansion of the *GhMLO* family was primarily driven by segmental and tandem duplications. Integration of transcriptomic data from the COTTONOMICS database revealed tissue-specific expression patterns, with higher transcript abundance in receptacles, stems, and roots, but lower levels in stamens and petals. Salt, drought, and cold stress treatments induced upregulation of *GhMLO* family members, with most genes showing increased expression over time. RT-qPCR analysis validated that five candidate *GhMLO* genes were significantly upregulated under salt stress. In summary, this study provides a comprehensive genome-wide characterization of the *GhMLO* gene family, elucidating their phylogenetic relationships and expression dynamics, which establishes a theoretical basis for identifying key regulatory genes involved in abiotic stress responses and offers novel genetic resources for improving stress tolerance in cotton molecular breeding.

## 1. Introduction

*Gossypium hirsutum*, the world’s primary source of natural fiber, holds dual strategic value for ecological restoration and rural revitalization in the saline-alkali regions of northern and northwestern China [1,2]. Salt stress significantly suppresses cotton growth, causing stunted development, leaf chlorosis, impaired root systems, and reductions in both yield and fiber quality [3,4]. Therefore, the discovery, identification, and utilization of salt-tolerant genes to enhance cotton’s salinity tolerance and improve the comprehensive utilization efficiency of saline-alkali lands holds profound implications [5,6].

A gene family comprises a set of homologous genes derived from a common ancestral gene through duplication and divergence, often encoding functionally similar or synergistic proteins that constitute a functional module within an organism [7,8,9]. The *MLO* (Mildew Resistance Locus O) gene family encodes specialized seven-transmembrane proteins initially identified as powdery mildew susceptibility factors in barley [10]. MLO proteins mediate broad spectrum resistance against fungal, bacterial, and viral pathogens by modulating apoplastic Ca^2+^ channel activity and ROS homeostasis [11]. Additionally, they participate in signal transduction pathways under abiotic stresses including salinity, drought, cold, and heavy metal toxicity and maintain cell wall integrity [12,13]. In *Arabidopsis*, mutations in *AtMLO2/6/12* confer complete resistance to powdery mildew, whereas loss of function mutants of *OsMLO12* exhibit male gametophyte sterility, with impaired pollen hydration and germination both in vitro and in vivo, preventing generation of homozygous progeny [14,15,16]. Although initially recognized for their role in regulating powdery mildew resistance, recent studies have demonstrated that *MLO* genes also play significant roles in abiotic stresses such as salt, drought, extreme temperature stress, etc. [14,17,18]. The function of *MLO* in abiotic stress can be summarized as “negatively regulating osmotic/oxidative stress tolerance and participating in stress-immune cross-signaling [19]. At present, the MLO protein family has been defined and explored in more than 40 plant species, including *Arabidopsis thaliana*, *Malus pumila*, *Citrullus lanatus* and *Gosspium* [20,21,22]. However, the role of *MLO* genes in responding to salt stress in cotton lacks systematic investigation.

A bHLH family member, designated *GhbHLH* (GenBank: KJ605396), from a normalized cDNA library of *G. hirsutum* cultivar JiMian 228. This gene encodes a 199 amino acid protein with a 600 bp open reading frame, shares 52% homology with *Arabidopsis AtbHLH149*, and contains a conserved basic helix-loop-helix (bHLH) domain. Expression of *GhbHLH* is induced by abiotic stresses such as high salinity and drought, showing maximal upregulation in roots that peaks at 2 h post treatment and remains significantly higher than in stems and leaves. *Agrobacterium*-mediated transformation generated *GhbHLH149-like* overexpression lines, and comparative transcriptomic analysis against recipient control JIN668 revealed significant upregulation of *GhMLO43* in transgenic lines.

Previously, we generated *GhbHLH149-like* overexpressing cotton lines via *Agrobacterium*-mediated transformation. Comparative transcriptome analysis against the recipient control JIN668 revealed significant upregulation of *GhMLO43* in transgenic lines, a finding further corroborated by RT-qPCR demonstrating consistent upregulation at multiple time points under salt stress. In this study, based on the latest genomic data, we identified 46 *GhMLO* members through a combination of hidden Markov models and iterative BLAST searches, clustering them into four subfamilies. To characterize the structural features of GhMLO proteins, transmembrane topology and conserved domain analyses were performed, and the results indicated that all *GhMLO* members harbor the conserved MLO domain as well as the typical transmembrane architectures that are characteristic of the MLO protein family. Comparative structural analysis revealed a high degree of similarity between GhMLO proteins and their homologs from dicotyledonous model plant species, suggesting evolutionary conservation of this gene family across dicot lineages. Collinearity assessment was also conducted to explore the evolutionary mechanisms underlying the expansion of the *GhMLO* gene family in *Gossypium hirsutum*, and the findings demonstrated that segmental duplication and tandem duplication are the primary driving forces responsible for the family’s expansion. Transcriptomic datasets obtained from the COTTONOMICS database were integrated and analyzed, and the results showed that *GhMLO* genes display obvious tissue-specific expression patterns: notably high expression levels were detected in vegetative tissues including leaves, stems, and roots, whereas expression was relatively weak in reproductive tissues such as stamens and petals. Additionally, abiotic stress treatments (salt, drought, and low temperature) were applied to explore the stress-responsive characteristics of *GhMLO* genes, and the results showed that numerous *GhMLO* family members were induced and up-regulated under these stress conditions, with the majority of these genes showing a gradual increase in expression level as the stress treatment duration prolonged. RT-qPCR results confirmed that all five selected candidate *GhMLO* genes exhibited significant up-regulation in response to salt stress. Collectively, the systematic identification and comprehensive analysis of the *GhMLO* gene family in *G. hirsutum* not only offer novel perspectives and potential targets for the screening of candidate *GhMLO* genes but also establish a solid basis for further deciphering their biological roles in cotton growth and development processes, as well as their regulatory mechanisms in mediating responses to abiotic stresses. In conclusion, this study provides crucial theoretical support for the identification of key regulatory genes involved in cotton abiotic stress adaptation and developmental regulation, which is expected to provide valuable guidance for subsequent research on cotton stress resistance breeding and genetic improvement.

## 2. Results

### 2.1. Identification, Phylogenetic Analysis and Sequence Alignment of the GhMLOs Gene Family

To systematically investigate the function of *GhMLO* gene family in *G. hirsutum*, we performed genome-wide identification and curation of family members. Following elimination of redundancies and integration of NCBI chromosome mapping information, we identified a total of 46 *GhMLO* genes (Supplementary Table S1). Physicochemical characterization revealed that the encoded proteins ranged from 85 to 584 amino acids (average 484.7 aa), with 85% exhibiting molecular weights exceeding 50 kDa. Predicted isoelectric points (pI) varied from 6.30 to 9.86, with three proteins classified as acidic and 44 as basic. 22 members displayed instability indices below 40, indicating high stability. 21 proteins were predicted to be hydrophilic, whereas the remainder were hydrophobic. Subcellular localization prediction revealed plasma membrane localization for 45 GhMLO proteins.

**Figure 1:**
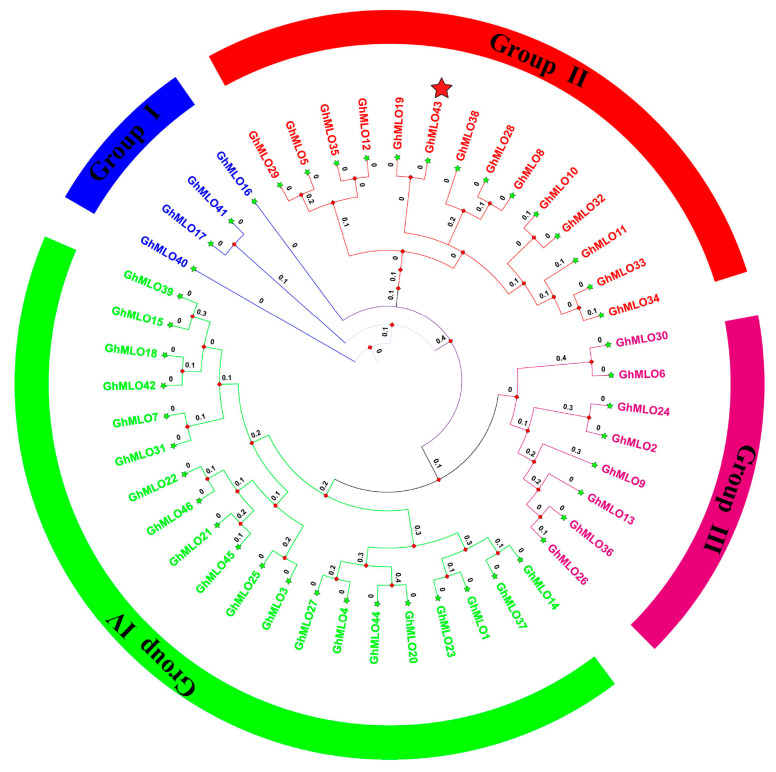
Systematically evolved tree of the *GhMLO* family proteins in *G. hirsutum*. The *G. hirsutum GhMLOs* gene family is divided into four subgroups based on branch color. The red star specifically marks GhMLO43.

**Figure 2:**
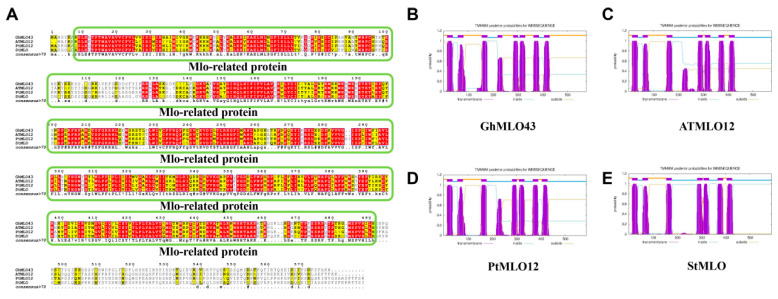
Analysis of the Properties of the *GhMLO43* Protein in *G. hirsutum. (***A**): Amino acid sequence alignment of *GhMLO43* with MLO proteins from three other species; green boxes indicate conserved domains of Mlo-related proteins; conserved amino acids are denoted by red. (**B**–**E**): Analysis of transmembrane domains in *GhMLO43*; *ATMLO12*; *PtMLO12*; *StMLO*.

Based on 46 *G. hirsutum GhMLO* amino acid sequences, a phylogenetic tree was constructed using the MEGA-X maximum likelihood method (ML). Phylogenetic analysis divided the *GhMLO* family into four distinct branches (Figure 1), each with different numbers of members, but sequences within the same branch were highly homologous (Figure 1). To further verify conservation, CLUSTALX multi-sequence alignment was performed on MLO proteins from four representative species (*G. hirsutum*, *Arabidopsis thaliana*, *Populus trichocarpa*, and *Solanum tuberosum*), and the results showed that all MLO proteins contained typical MLO conserved domains and seven transmembrane helical regions (Figure 2A–E). To sum up, the *GhMLO* gene family is highly conserved during evolution, and its structural and functional characteristics are common between *G. hirsutum* and other species.

### 2.2. Chromosomal Distribution and Collinearity Analysis of the GhMLOs Gene Family

**Figure 3:**
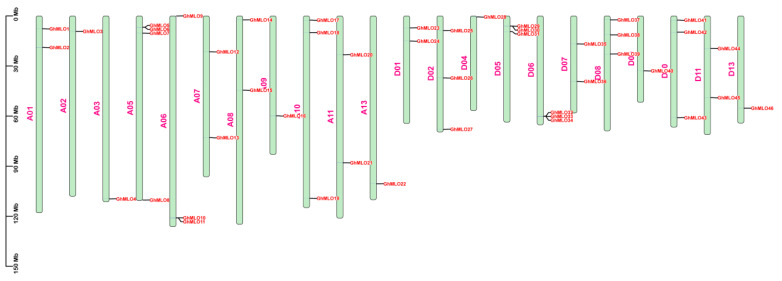
Chromosomal localisation of the *GhMLO* gene family in *G. hirsutum.* The scale on the left is marked in 30 Mb increments. The sequence number of each chromosome is displayed on the left side of each chromosome. The gene names on the right side of each chromosome correspond to the position of each *GhMLO* gene.

**Figure 4:**
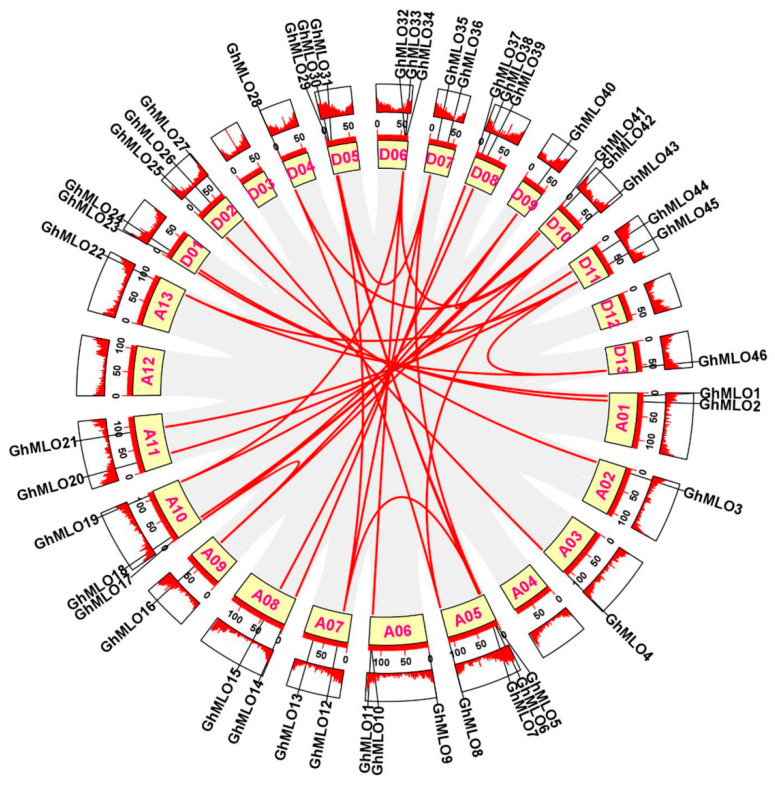
Intraspecific collinearity analysis of the *GhMLO* gene family in *G. hirsutum. *The size of the fan-shaped rings represents the chromosome length of *G. hirsutum*. The gray blocks in the background indicate collinear blocks within the genome, while the red lines denote homologous gene pairs of *GhMLOs*.

**Figure 5:**
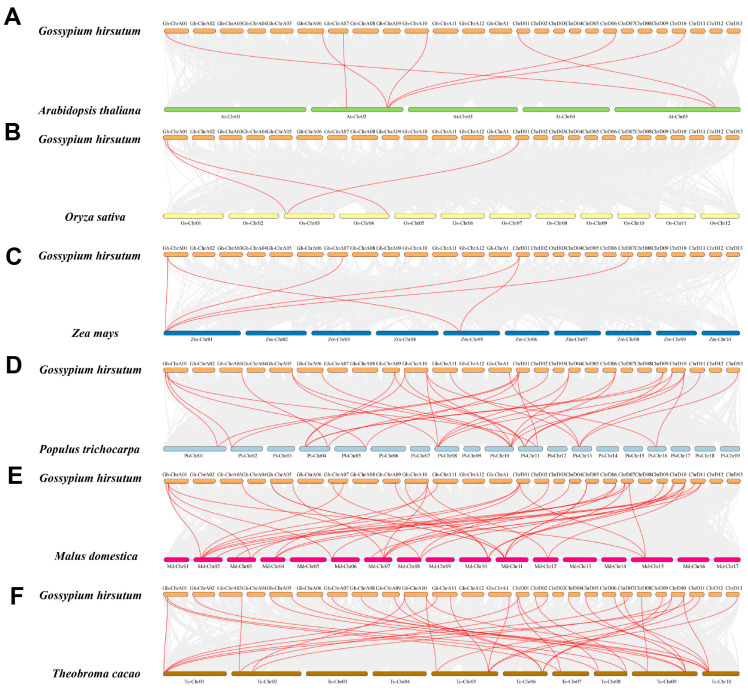
Interspecies colinearity analysis of the *GhMLO* gene family in *G. hirsutum*. (**A**): *Arabidopsis thaliana*; (**B**): *Oryza sativa*; (**C**): *Zea mays*; (**D**): *Populus trichocarpa*; (**E**): *Malus domestica*; (**F**): *Theobroma cacao*. Gray lines in the background indicate collinear blocks between the genome of *G. hirsutum* and those of other species, while red lines highlight the collinear *GhMLO* gene pairs.

Chromosome localization results showed that 46 *GhMLO* genes were distributed across 22 chromosomes of *G. hirsutum* (Figure 3). Among them, chromosome A05 carries the largest number of members (4), while the rest of the chromosomes contain 1–3 genes. 4 *GhMLO* tandem repeat clusters *(GhMLO5/6, GhMLO10/11*, *GhMLO29/30* and *GhMLO32/33/34*) were detected on chromosomes A05, A06, D05 and D06. Intraspecific collinearity analysis further identified 33 pairs of *GhMLO* fragment duplication events (Figure 4). To analyze cross-species evolutionary relationships, collinearity comparisons of *G. hirsutum* were conducted with *Arabidopsis thaliana*, rice, corn, *G. hirsutum*, apple, and cocoa, resulting in 7, 3, 6, 35, 36, and 37 homologous gene pairs (Figure 5A–F). The results suggest that the *GhMLO* family has a closer genetic relationship with woody plants in terms of evolution.

### 2.3. Analysis of the Gene Structure and Protein Conserved Motifs of the GhMLOs Gene Family

**Figure 6:**
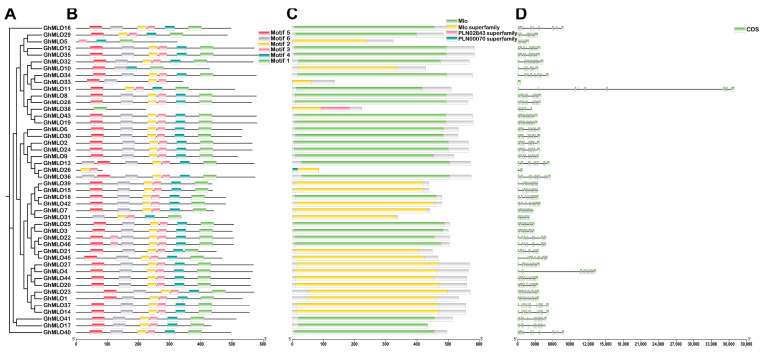
Evolutionary analysis (**A**), conserved motifs (**B**), conserved domains (**C**), and gene structure (**D**) analysis of the *GhMLO* gene family in *G. hirsutum.* Motif analysis of the *GhMLO* gene family, with individual motifs displayed as squares in different colors. Exons are represented by green rectangles, while introns are indicated by gray lines.

To clarify the evolutionary relationships of the *GhMLO* gene family, we integrated phylogenetic trees, conserved motifs, protein domains and gene structures for multi-dimensional analysis (Figure 6A–D). Six conserved motifs were identified from 46 GhMLO protein sequences. The number and distribution of motifs varied, but the family as a whole was highly conserved: all members contained at least two motifs, with Motif-3 present in all 46 proteins, forming a characteristic marker of the MLO domain. Domain analysis revealed that all GhMLO proteins contained the MLO core domain. Notably, *GhMLO38* and *GhMLO26* also carry two additional domains, PLN02843 and PLN00070. In terms of genetic structure, except for *GhMLO33* which is a single exon gene, the rest of the members have introns, with the number of exons ranging from 1 to 16.

### 2.4. Analysis of Cis-Acting Elements in the GhMLOs Gene Family Promoter

**Figure 7:**
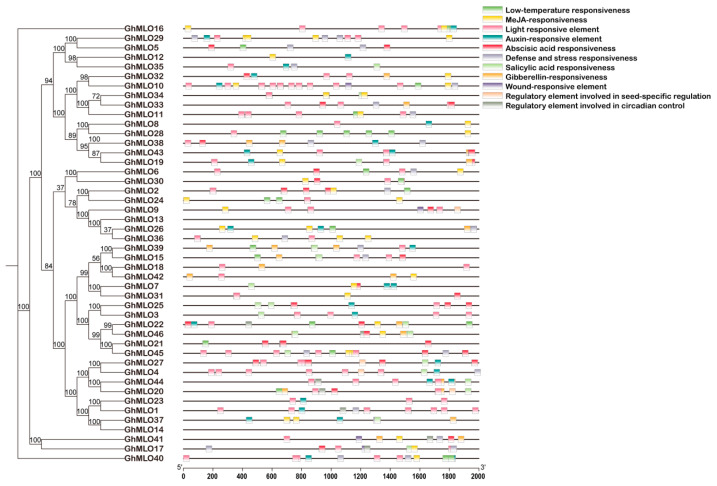
Analysis of cis-acting elements in the promoters of the *GhMLO* gene family in *G. hirsutum*. Rectangular blocks of different colors represent different response components.

To analyze the expression regulatory characteristics of the *GhMLOs* gene family, the cis-acting elements in its promoter region were systematically predicted using the PlantCARE database. The results showed that all *GhMLOs* promoters contained photoresponsive elements, and most members carried hormone regulatory elements in response to methyl jasmonate (MeJA), abatinic acid (ABA), gibberellin (GA), salicylic acid (SA), and auxin (IAA). In addition, elements related to the low-temperature response, circadian rhythm, defense and stress response were also widely present (Figure 7). There are significant differences in the types and quantities of cis-type elements among different members, suggesting that the *GhMLOs* family has diverse and specific regulatory functions in *G. hirsutum* growth and development and stress response.

### 2.5. Expression Patterns of the GhMLOs Gene Family

**Figure 8:**
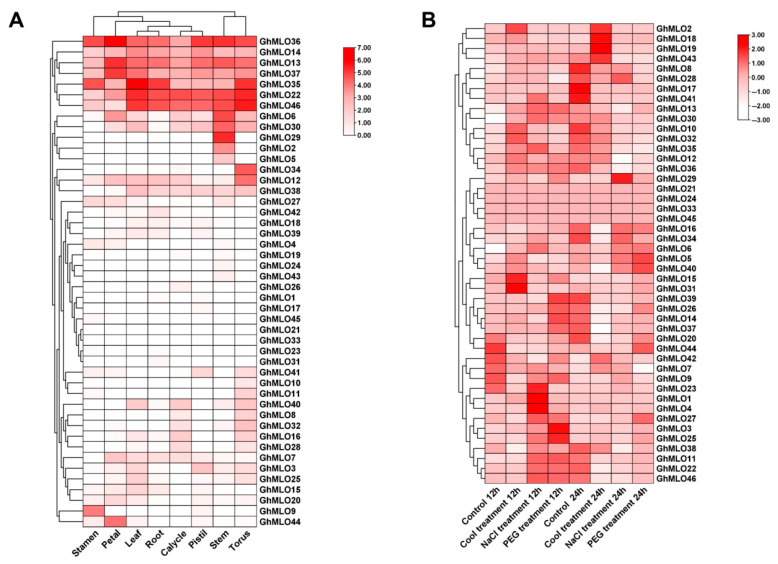
Heatmap of transcriptomic analysis for the *GhMLO* gene family in *G. hirsutum* across different tissues **(A)** and under various abiotic stresses **(B).** The color bar indicates the range of maximum and minimum expression levels in the heatmap, with varying shades of red representing high, medium, and low expression levels.

To further clarify the role of the *GhMLOs* gene family in cotton growth and development and abiotic stress, this study obtained expression profiles of eight representative tissues and three abiotic stresses (cold, simulated drought, salt) from the COTTONOMICS transcriptome database (Figure 8A,B). The tissue expression profiles showed that the vast majority of *GhMLOs* were highly expressed in receptacles, such as *GhMLO22*, *GhMLO35* and *GhMLO46*; *GhMLO36* was maintained at high levels in all eight tissues, suggesting that it may play an important regulatory role in the overall growth and development of cotton. Stress expression profiles indicated that *GhMLO1, GhMLO4*, *GhMLO23*, etc., were significantly upregulated under salt stress, while *GhMLO15*, *GhMLO31*, *GhMLO43*, etc., were enhanced under cold stress, revealing that related members of the gene family were respectively involved in the response processes of salt stress and cold stress.

### 2.6. Expression of GhMLOs Under Salt Stress

**Figure 9:**
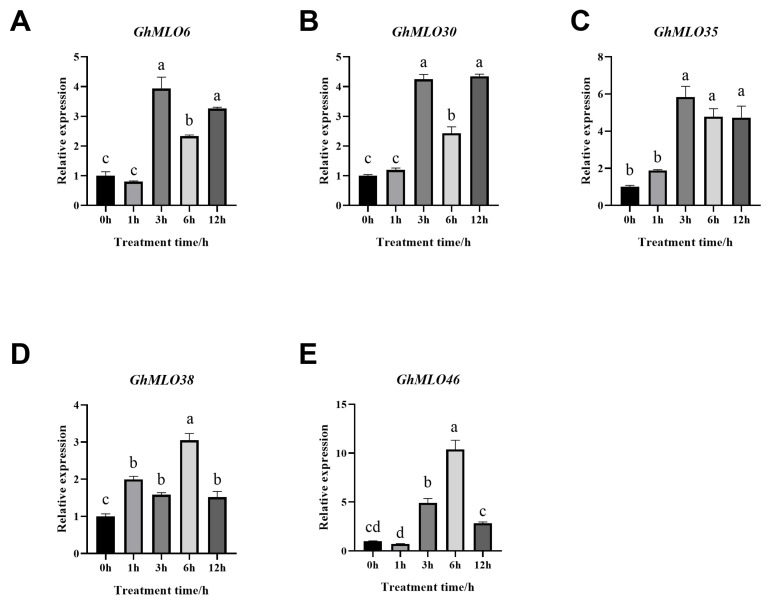
Expression analysis of *GhMLOs* under salt stress treatment. (**A**): *GhMLO6*; (**B**): *GhMLO30*; (**C**): *GhMLO35*; (**D**): *GhMLO38*; (**E**): *GhMLO46*. One-way ANOVA with LSD multiple comparison, n = 12, Significant differences (*p* < 0.05) are indicated by columns marked with distinct letters..

The *GhMLO* gene family encodes transmembrane proteins whose expression is regulated by ABA, ROS, and temperature signaling networks, making it an important node for studying stress-immune cross-responses. The expression levels of five *GhMLO* genes at different time points under normal conditions and salt stress were detected by RT-qPCR to verify the role of these genes under salt stress. The results showed that all five *GhMLO* genes were upregulated under salt stress treatment, but the response times were different (Figure 9A–E). The results showed that the *GhMLO* gene families in *G. hirsutum* responded differently to salt stress treatment.

## 3. Discussion

A total of 46 *GhMLO* genes were identified in this study. Phylogenetic and motif analyses revealed that all members contained seven transmembrane domains and calmodulin binding domains (CaMBD) at the C-end, showing typical basic isoelectric point characteristics. Subcellular localization predictions indicated that they were mainly located on the plasma membrane (Figure 6B,C, Supplementary Table S1). The CaMBD conserved domain gives GhMLO protein the ability to interact dynamically with calmodulin (CaM), thereby mediating calcium signal transduction in response to external stress [23,24]. It is notable that the *GhMLO* gene family typically contains multiple introns, and given that intron-free genes usually have higher transcriptional efficiency, it is speculated that the presence of introns may give *GhMLO* transcriptional dynamic characteristics of delayed regulation or fine regulation under adverse conditions (Figure 6D) [25]. *GhMLO* genes in different plants are highly homologous, and comparisons of their amino acid sequences suggest that they are evolutionarily conserved. The mechanisms of gene family growth and genome evolution are largely dependent on gene duplication events, including whole genome duplication (WGD), segmental duplication (SD), and tandem duplications (TD), etc. [26]. In this study, 46 *GhMLO* genes were distributed across 22 chromosomes, including 33 SD gene pairs and 4 TD gene clusters (Figure 3 and Figure 4). Gene pairs of gene replication events may have similar functions and expression patterns. For example, the highly homologous gene pairs *GhMLO5/6* and *GhMLO10/11* formed through series replication have similar transcriptional levels under different stress conditions and in different tissues, respectively (Figure 8A,B).

Although the functions of the MLO protein family have been identified in many species, there are certain differences in the functions among different species [17]. The MLO protein inhibits the deposition of antibacterial substances such as callose by directly interacting with SNARE protein ROR2 and blocking the directional transport of disease-resistant vesicles to the infection site [27,28]. At the same time, the interaction also downregulates the transcriptional levels of the glycoside hydrolase PEN2 and the ABC transporter PEN3, weakening the plant’s ability to degrade and excrete mycotoxins [29]. *CML45* (calmodulin protein), *ZTP29* (zinc transporter), *LSD1* (zinc finger protein), and *MYB* in Goji berries may specifically interact with TC-rich repeats to regulate, thereby coordinating the expression of the *LbMLO2* gene to enhance goji berries’ resistance to powdery mildew [30,31,32].

It participates in abiotic stress responses in a variety of species such as melons, rice and peppers. The analysis in this study showed that the vast majority of *GhMLO* genes were significantly upregulated under simulated conditions of drought, low temperature and salt stress (Figure 8B). We further conducted a systematic analysis of the *GhMLO* gene family promoter sequences and found that they were enriched with a variety of cation-acting elements associated with abiotic and biological stresses, such as ABRE and ERE (Figure 7) [33]. At 6 h of salt stress and 12 h of drought stress in bitter gourd, the relative expression of *McMLO11* in bitter gourd leaves reached its peak, 56.7 and 9.07 times that of the control, respectively, suggesting that the *McMLO11* gene can respond positively to drought and salt induction [34]. We treated the *HX1* strain with 200 mM salt stress and found that all 5 *GhMLO* genes were upregulated under salt stress and may play an important role in resisting salt stress (Figure 9). *bHLH* (basic helix-loop-helix) transcription factor is one of the core nodes in the plant salt tolerance regulatory network, and *bHLH* can enhance plant salt tolerance by eliminating reactive oxygen species (ROS), maintaining ionic homeostasis, and hormone signaling crossover. The *GhbHLH149-like* gene in *G. hirsutum* plays a significant role in salt tolerance stress, and overexpression can enhance salt tolerance in cotton. The expression of *GhMLO43* in overexpressed *GhbHLH149-like* cotton plants was significantly higher than that in wild-type cotton, and the expression of *GhMLO43* was also significantly upregulated under salt stress, indicating that *GhMLO43* functions under salt stress and may be regulated by *GhbHLH149-like* expression (Figure S1A,B).

Soil salinization is a major limiting factor for global agricultural production. The core mechanism by which plants respond to salt stress depends on the SOS pathway [3]. Salt stress induces an increase in cytoplasmic Ca^2+^, the calcium sensor *CBL4/CBL10* binds and activates the kinase *CIPK24* (*SOS2*), and then phosphorylates the Na^+^/H^+^ reverse transporter *SOS1* for Na^+^ efflorescence or vacuole fragmentation [35,36]. Enhanced salt tolerance. *MLO* (such as *AtMLO1/5/9/15*) may be a type of calcium channel specific to plants that mediates extracellular Ca^2+^ influx and serves as a core hub for Ca^2+^ signaling in plant polar growth [37]. However, the mechanism by which the *GhMLO* gene regulates plant salt tolerance through the Ca^2+^ signaling pathway is still understudied. It can be speculated that clarifying the function of the *GhMLO* gene in the salt tolerance response of *G. hirsutum* may be of great significance. This study systematically identified members of the *GhMLO* gene family using bioinformatics methods and screened out multiple candidate proteins involved in the salt stress response. Compared with previous studies [22], we identified more *GhMLO* genes by utilizing updated genome data, and performed comprehensive analyses on their gene structure, protein conserved motifs, cis-acting elements, as well as the expression levels of some members at different response time after salt stress treatment. In summary, the *GhbHLH149-like—GhMLO43* signaling module may play a key role in salt tolerance of *G. hirsutum* by regulating calcium ion signal transduction. *GhMLO43* is currently the only member with identified regulatory context, and future studies will explore the functional roles of other candidates.

## 4. Materials and Methods

### 4.1. Cotton Test Materials

The cotton materials used in this study were the *JIN668* and *HX1* lines. Among these, *HX1* is a multi-year selected line developed by our laboratory, exhibiting excellent comprehensive traits, strong drought resistance, and broad adaptability. *JIN668* was provided by Professor Jin Shuangxia of Huazhong Agricultural University and serves as a commonly used model recipient in cotton genetic transformation research. The *GhbHLH149-like* overexpression vector was used to transform *JIN668* by *Agrobacterium tumefaciens*-mediated transformation to obtain *GhbHLH149*-like overexpressing cotton lines. Cotton seeds were soaked in water for 12–18 h before being inoculated into nutrient soil (nutrient soil:vermiculite 1:1). After two weeks, uniformly growing seedlings were selected for salt stress treatment (200 mM NaCl solutions were supplemented with nutrient solution). The treatment was performed with three biological replicates. Five plants were mixed for RNA extraction per sample, which was subsequently used for qRT-PCR data analysis [2].

### 4.2. Identification of the GhMLOs Gene Family in G. hirsutum and Analysis of Their Protein Properties

Using the HMMER 3.0 software, candidate gene family members were identified by performing homology searches in the *G. hirsutum* reference genome using the MLO protein hidden Markov model (PF03094) provided by the Pfam 35.0 database. The *GhMLO* gene family was confirmed using the protein BLAST database (https://blast.ncbi.nlm.nih.gov/Blast.cgi (accessed on 4 August 2025)). The *G. hirsutum* TM-1 reference genome (v2.1), transcripts, amino acids, and 2000 base pairs upstream of the ATG promoter region were extracted from the COTTONOMICS database (http://cotton.zju.edu.cn/ (accessed on 4 August 2025)) [38]. *GhMLOs* were named according to their chromosomal positions (*GhMLO1*-*GhMLO46*). *GhMLO* gene positions and chromosome sizes were obtained from the COTTONOMICS database and visualized using TBtools -II (South China Agricultural University, Guangzhou, China) [39]. Protein physicochemical property predictions were performed using the ProtParam website (https://web.expasy.org/protparam/ (accessed on 4 August 2025)). Subcellular localization predictions for the *GhMLO* gene family were conducted via the Plant-mPLoc website (http://www.csbio.sjtu.edu.cn/bioinf/plant-multi/ (accessed on 4 August 2025)).

### 4.3. Sequence Alignment and Phylogenetic Analysis of the GhMLOs Gene Family

Using MEGA7.0 software, a phylogenetic tree of the *GhMLOs* gene family was constructed via the Neighbor-Joining (NJ) method. Branch reliability was assessed through 1000 bootstrap resampling runs [40]. The resulting tree file was further imported into the iTOL online platform (https://itol.embl.de/ (accessed on 20 August 2025)) for advanced visualization, clearly illustrating its evolutionary relationships.

The amino acid sequences of the *ATML012*, *PtMLO12*, and *StMLO* were obtained from the Phytozome14 database (https://phytozome-next.jgi.doe.gov/ (accessed on 20 August 2025)). Sequence comparisons and result visualization were performed using the ClustalW website (https://www.genome.jp/tools-bin/clustalw (accessed on 20 August 2025)) and ENDscript/ESPrip (https://espript.ibcp.fr/ESPript/ESPript/index.php (accessed on 20 August 2025)). Additionally, the protein structures of these four genes were predicted using the TMHMM-2.0 website (https://services.healthtech.dtu.dk/services/TMHMM-2.0/ (accessed on 20 August 2025)) [41].

### 4.4. Analysis of the GhMLOs Gene Family: Conserved Motifs, Conserved Domains, and Gene Structure

To analyze the structural characteristics of the *GhMLOs* gene family, we first imported a file containing all *GhMLOs* gene ID and gff3 annotation files into TBtools for visualizing their exon-intron layouts. Subsequently, GhMLO protein sequences were submitted to MEME Suite (https://meme-suite.org/meme/index.html (accessed on 25 October 2025)). Under default parameters, the motif count was set to 6 to generate corresponding MEME results. The obtained protein sequences were simultaneously uploaded to the NCBI protein database to retrieve Hitdata files. Finally, the three datasets were integrated within the TBtools platform to jointly construct a phylogenetic tree, a conserved motif map, and a gene structure diagram for the *GhMLOs* family. This enabled a comprehensive analysis of the family at the sequence, evolutionary, and structural levels [42].

### 4.5. Collinearity Analysis of the GhMLOs Gene Family

Using TBtools with default parameters, we performed MCScanX analysis on the *G. hirsutum* genome’s FAST and gff3 files. We further assessed collinearity between *G. hirsutum* and other species. We employed the default parameters of TBtools’ One Step MCScanX function to identify potential homologous genes. We compared the genomic FAST and gff3 files of *G. hirsutum* with those of other species, obtaining three key files: the genomic gff file, the CTL file (control file), and the Collinearity file. First, we manually removed unnamed chloroplast and mitochondrial sequences from the CTL file, then reordered the data, and finally visualized it using TBtools [43].

### 4.6. Analysis of Promoter Cis-Acting Elements in the GhMLOs Gene Family

The cis-acting elements within the 2000 bp upstream region of the ATG promoter for the *GhMLOs* gene family were predicted using the PlantCARE website (http://bioinformatics.psb.ugent.be/webtools/plantcare/html/ (accessed on 25 October 2025)), and the results were visualized with TBtools.

### 4.7. Transcriptome Analysis of the GhMLOs Gene Family

To systematically analyze the expression characteristics of the *GhMLOs* gene family, this study downloaded the corresponding transcriptome dataset from the COTTONOMICS public database (http://cotton.zju.edu.cn/index.htm (accessed on 30 October 2025)). This dataset encompasses FPKM expression matrices from eight different tissues (root, stem, leaf, etc.) of *G. hirsutum* and three abiotic stress treatments (salt, simulated drought, cold). Subsequently, the “HeatMap” function module in TBtools software was used to generate an expression heatmap. Row scale normalization was applied with all other parameters set to default values to visually illustrate the differential expression patterns of *GhMLOs* across different tissues and stress conditions.

### 4.8. RT-qPCR Analysis of the GhMLOs Gene Family

RNA extraction was performed using the Nuoto^®^ AutoExtracter-32 Nucleic Acid Extractor with the 5fz PCR DNA/RNA AutoPurification Kit from Kangma-Healthcode (Shanghai, China). Subsequently, first-strand cDNA was synthesized using the AT311 Reverse Transcription Kit (TransGen, Beijing, China). Specific primers were designed using the NCBI Primer-BLAST online tool (Supplementary Table S2). RT-qPCR was performed on a CFX96 Touch™ real-time fluorescent quantitative PCR instrument (Bio-Rad, Co. Ltd., Hercules, CA,USA) using the SYBR Green detection system. Reaction program: 95 °C pre-denaturation for 15 min; 45 cycles of 95 °C denaturation for 10 s, 60 °C annealing for 30 s, and 72 °C extension for 30 s [44]. Relative expression levels were calculated using the 2^−ΔΔCT^ method, with *GhUBQ7* (*Ghir_D12G021700*) as the reference gene [2], and four technical replicates to ensure accuracy.

### 4.9. Statistical Analyses

We analyzed RT-qPCR experimental data using GraphPad Prism 8(GraphPad Software, Inc., Boston, Massachusetts, USA). For relative gene expression levels, we performed one-way analysis of variance (ANOVA) with LSD multiple comparison tests (*p* < 0.05). Prior to ANOVA, data were tested for normality and homogeneity of variance [45].

## 5. Conclusions

The *MLO* gene family is a plant-specific gene family with seven transmembrane domains and carboxyl-terminal calmodulin binding domains. The plant *GhMLO* gene is integrated and regulated by ABA, ROS, and temperature signaling networks, making it an important node for studying stress-immune cross-responses. By genomic and bioinformatics methods, 46 *GhMLO* genes in the *MLO* (Mildew resistance Locus O) gene family of *G*. *hirsutum* were classified and identified in this study. Chromosomal localization, evolutionary tree, gene structure, gene doubling, promoter cis-element, and expression profile of *GhMLO* were analyzed. Members of the *GhMLO* family could be significantly induced to expression by drought, low-temperature stress and salt stress, suggesting that *GhMLO* may play an important role in the resistance of *G. hirsutum* to abiotic stress. This study represents a preliminary investigation into the *GhMLO* gene family in *G. hirsutum*. The results provide candidate genes and a basis for future studies exploring their possible roles in abiotic stress adaptation, which could ultimately contribute to the development of cotton varieties with enhanced tolerance to salt, drought, and temperature stresses. Expression levels of the *GhMLO43* gene in *GhbHLH149-like* overexpression lines and under salt stress. 

## Data Availability

All data supporting the conclusions of this paper are provided in the article and its additional files. Genome sequence data for all species are available in the Phytozome database (https://phytozome-next.jgi.doe.gov/ (accessed on 4 August 2025)) and CottonFGD database (https://cottonfgd.net/ (accessed on 4 August 2025)). Publicly available RNA-seq data are available on CottonFGD database.

## Supplementary Materials

The following supporting information can be downloaded at: https://www.mdpi.com/article/10.3390/life16030476/s1, Table S1: List of *Gossypium_hirsutum GhGRF* genes. Table S2: Primers used for RT-qPCR in the article. Figure S1. Expression levels of the *GhMLO43* gene in *GhbHLH149-like* overexpression lines and under salt stress. (A) Expression heatmap of the *GhbHLH149-like* overexpression line *GhMLO43*. The color bar indicates the range of maximum and minimum expression values in the heatmap. (B) RT-qPCR analysis of *GhMLO43* expression levels under salt stress treatments at different time points. One-way ANOVA with LSD multiple comparison, n = 12, *p* < 0.05.
