# Supplementary material for: Identification and Expression Analysis of the MLO Gene Family Under Salt Stress in Cotton (Gossypium hirsutum L.)
Source: Life (Basel). 2026 Mar 16;16(3):476. doi: 10.3390/life16030476 (PMC13028013; doi:10.3390/life16030476)
Supplement: Supplementary file 1 [file life-16-00476-s001.zip › Figure S1. Expression levels of the GhMLO43 gene in GhbHLH149-like overexpression lines and under salt stress..pdf]

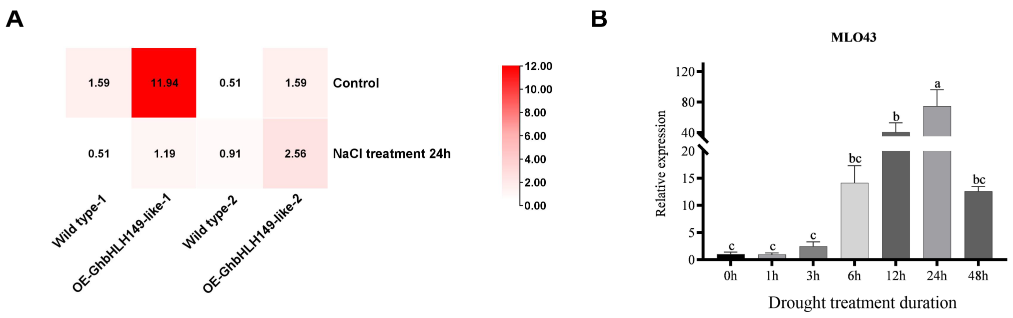

Figure S1. Expression levels of the *GhMLO43* gene in *GhbHLH149-like* overexpression lines and under salt stress. A: Expression heatmap of the *GhbHLH149-like* overexpression line *GhMLO43*. The color bar indicates the range of maximum and minimum expression values in the heatmap. B: RT-qPCR analysis of *GhMLO43* expression levels under salt stress treatments at different time points. One-way ANOVA with LSD multiple comparison, n = 12, $p < 0.05$.
